# Blockade of BDNF signalling attenuates chronic visceral hypersensitivity in an IBS‐like rat model

**DOI:** 10.1002/ejp.1534

**Published:** 2020-02-05

**Authors:** Fei Fan, Ying Tang, Hengfen Dai, Yang Cao, Pei Sun, Yu Chen, Aiqin Chen, Chun Lin

**Affiliations:** ^1^ School of basic Medical Sciences Laboratory of Pain Research Fujian Medical University Fuzhou China; ^2^ Fujian Provincial Key Laboratory of Brain Aging and Neurodegenerative Diseases Fujian Medical University Fuzhou China; ^3^ Affiliated Fuzhou First Hospital of Fujian Medical University Fuzhou China; ^4^ Present address: Fujian Health College Fuzhou China

## Abstract

**Background:**

Irritable bowel syndrome (IBS) is a common functional disease characterized by chronic abdominal pain and changes in bowel movements. Effective therapy for visceral hypersensitivity in IBS patients remains challenging. This study investigated the roles of brain‐derived neurotrophic factor (BDNF) and tyrosine kinase receptor B (TrkB) and the effect of ANA‐12 (a selective antagonist of TrkB) on chronic visceral hypersensitivity in an IBS‐like rat model.

**Methods:**

An IBS‐like rat model was established through neonatal maternal separation (NMS), and visceral hypersensitivity was assessed by electromyographic (EMG) responses of the abdominal external oblique muscles to colorectal distention (CRD). Different doses of ANA‐12 were injected intrathecally to investigate the effect of that drug on visceral hypersensitivity, and the open field test was performed to determine whether ANA‐12 had side effects on movement. Thoracolumbar spinal BDNF, TrkB receptor and Protein kinase Mζ (PKMζ) expression were measured to investigate their roles in chronic visceral hypersensitivity. Whole‐cell recordings were made from thoracolumbar superficial dorsal horn (SDH) neurons of lamina II.

**Results:**

The expression of BDNF and TrkB was enhanced in the thoracolumbar spinal cord of the NMS animals. ANA‐12 attenuated visceral hypersensitivity without side effects on motricity in NMS rats. PKMζ expression significantly decreased after the administration of ANA‐12. The frequency of spontaneous excitatory postsynaptic currents (sEPSCs) increased in the thoracolumbar SDH neurons of lamina II in NMS rats. The amplitude and frequency of sEPSCs were reduced after perfusion with ANA‐12 in NMS rats.

**Conclusions:**

Neonatal maternal separation caused visceral hypersensitivity and increased synaptic activity by activating BDNF‐TrkB‐PKMζ signalling in the thoracolumbar spinal cord of adult rats. PKMζ was able to potentiate AMPA receptor (AMPAR)‐mediated sEPSCs in NMS rats. ANA‐12 attenuated visceral hypersensitivity and synaptic activity by blocking BDNF/TrkB signalling in NMS rats.

**Significance:**

ANA‐12 attenuates visceral hypersensitivity via BDNF‐TrkB‐PKMζ signalling and reduces synaptic activity through AMPARs in NMS rats. This knowledge suggests that ANA‐12 could represent an interesting novel therapeutic medicine for chronic visceral hypersensitivity.

## 1 INTRODUCTION

Irritable bowel syndrome (IBS) is a chronic, functional disease, characterized by the presence of chronic abdominal pain, bloating and changes in bowel habits; IBS affects 11% of the world's population (Lacy et al., 2016) and imposes a significant socioeconomic burden (Canavan, West, & Card, 2014; Deiteren, 2016). The pathophysiology of IBS involves visceral hypersensitivity, psychological disorders and altered intestinal motility (Drossman, Camilleri, Mayer, & Whitehead, 2002; Kanazawa, Hongo, & Fukudo, 2011; Melchior, Bril, Leroi, Gourcerol, & Ducrotté, 2018). However, the underlying mechanisms of visceral hypersensitivity in IBS patients have not yet been fully elucidated, and there is still no satisfactory treatment at present. Thus, the search for effective therapeutic strategies against IBS remains a significant challenge.

Visceral hypersensitivity is related to both central and peripheral sensitization (Lin & Al‐Chaer, 2003). Long‐term potentiation (LTP) of synaptic strength could be one of the mechanisms underlying central sensitization (Ji, Kohno, Moore, & Woolf, 2003; Sandkühler, 2007). Brain‐derived neurotrophic factor (BDNF) and protein kinase Mζ (PKMζ), two of the molecules we examine in this study, critically contribute to LTP, memory and pain (Ji et al., 2003; Melemedjian et al., 2013; Price & Ghosh, 2013; Sacktor & Hell, 2017). Overexpression of BDNF has been linked to bladder inflammation, and the Val66Met mutation of BDNF can affect pain processing at the cortical level (Coelho, Oliveira, Antunes‐Lopes, & Cruz, 2019). Recent studies have shown that BDNF contributes to visceral hypersensitivity in the colon (Fu et al., 2018; Zhang, Qin, Liu, Wang, & Yao, 2019). Peripheral and central BDNF and tyrosine kinase receptor B (TrkB; the selective receptor for BDNF) are involved in chronic and neuropathic pain (Minichiello, 2009; Smith, 2014). ANA‐12 (N‐[2‐[[(hexahydro‐2‐oxo‐1H‐azepin‐3‐yl)amino]carbonyl]phenyl]‐benzo[b]thiophene‐2‐carboxamide) has been identified as a selective TrkB antagonist and has been shown to relieve allodynia and visceral hypersensitivity (Burgos‐Vega, Quigley, Avona, Price, & Dussor, 2017; Fu et al., 2018; Liu et al., 2018). However, the roles of BDNF/TrkB and ANA‐12 in the spinal cord of IBS model rats remain controversial and need to be further explored. We hypothesize that BDNF/TrkB might play a key role in visceral hypersensitivity and that ANA‐12 possibly attenuates visceral hypersensitivity in the thoracolumbar spinal cord of adult IBS model rats.

Protein kinase Mζ (PKMζ), similar to BDNF, plays an important role in the maintenance of LTP, pain plasticity and long‐term memory (Price & Ghosh, 2013; Sacktor & Hell, 2017). Inhibiting PKMζ in the anterior cingulate cortex alleviated neuropathic pain (Ko et al., 2018; Li et al., 2010). Previously, we found that zeta inhibitory peptide (an inhibitor of PKMζ) could attenuate chronic visceral hypersensitivity in rats subjected to neonatal maternal separation (NMS; Tang et al., 2016); PKMζ is an atypical specific protein kinase C that is involved downstream of phospholipase Cγ1, in one of the three main intracellular signalling cascades activated by the TrkB (Huang & Reichardt, 2003; Reichardt, 2006). BDNF and PKMζ compensate for each other to maintain LTP (Sajikumar & Korte, 2011). However, little is known about the exact relationship between BDNF and PKMζ in NMS rats.

In this study, an IBS rat model was established by NMS on postnatal days 3–21. Visceral hypersensitivity was assessed by measuring the amplitude of electromyographic (EMG) responses to colorectal distention (CRD). The expression of BDNF, TrkB and PKMζ at the thoracolumbar spinal cord was measured by western blotting. The effects of ANA‐12 on visceral hypersensitivity were evaluated. Excitatory synaptic transmission at superficial dorsal horn (SDH) neurons in the lamina II of the thoracolumbar spinal cord was recorded by whole‐cell patch clamp.

## 2 MATERIALS AND METHODS

### 2.1 Animals

Male Sprague–Dawley rats were provided from the Department of the Experimental Animal Center of Fujian Medical University. NMS was established as described previously (Tang et al., 2016). Briefly, on postnatal 3–21 days neonates were separated from their mothers for 180 min every day. Single young rat was placed in a little compartment with sawdust bedding in different room. Control rats stayed with their mothers. The tests were performed when the experimental rats were 7–8 weeks old. Animal procedures were approved by the Committee for Care and Use of Laboratory Animals at Fujian Medical University. The study had a single‐blind cross‐sectional experimental design.

### 2.2 Assessment of visceral hypersensitivity

Electromyographic measurements were carried out as described previously to assess visceral hypersensitivity (Tang et al., 2016). Briefly, male rats (7–8 weeks old) were anaesthetized with isoflurane. A balloon was inserted into the colorectum, and the attached tube was secured to the rat's tail. Silver bipolar electrodes were inserted into the external oblique muscle of the abdomen. The balloon pressure was increased to 40 or 60 mmHg and sustained at that pressure for 10 s at intervals of 4 min. EMG responses to different degrees of CRD were recorded three times and collected with an RM6240BD system (Chengdu, China). Data were analysed after the mean baseline amplitude was subtracted; the average increase over baseline was used to assess visceral hypersensitivity.

### 2.3 Intrathecal catheter implantation and agent administration

The rats were anaesthetized with barbanylum (8%, 0.1 ml/100 g). A sterile polyethylene catheter (BB31695‐PE/1, Scientific Commodities Inc) was inserted between the L6 and S1 vertebrae to reach the lumbar enlargement. Rats showing neurological deficits after catheter implantation were euthanized. The rats were omitted from the experiment if hind paw paralysis did not occur after intrathecal injection of lidocaine (Maixner, Yan, Gao, Yadav, & Weng, 2015). Agents were administered 1 week after cannulation. Various dosages (20, 40 and 80 μg) of ANA‐12 (first dissolved in 100% DMSO to 8 mg/ml, which was then diluted to 1% DMSO with normal saline) were administered in a volume of 10 µl.

### 2.4 Open field test

To assess the effects of intrathecal ANA‐12 injections on spontaneous motor activity, we administered the open field test 30 min after intrathectal injection. A grey‐walled box (100 × 100 × 60 cm) with a black floor and an open top was prepared in a quiet room. A single rat was gently placed on the floor in the centre of the box, and an overhead camera immediately recorded and tracked the movements of the animal for 5 min. The total walking distance (m) and average speed (cm/s) were analysed. After every test, the box was carefully cleaned to remove any residue that could serve as a cue to the next rat.

### 2.5 Western blotting

The expression of thoracolumbar spinal cord BDNF, TrkB and PKMζ in rats was measured by western blotting. A total of 30μg of protein per sample was separated by electrophoresis and transferred to polyvinylidene fluoride (PVDF) membranes (Invitrogen, USA). The following antibodies were used: rabbit anti‐BDNF polyclonal antibody (BDNF, NB100‐98682; NovusBio); rabbit anti‐TrkB polyclonal antibody (ab18987; Abcam); rabbit anti‐PKCζ monoclonal antibody (ab59364, Abcam); rabbit anti‐GAPDH polyclonal antibody (Cat. No. AP0063, Bioworld Technology Inc.); mouse anti‐β‐tubulin monoclonal antibody (Cat. No. EM31013‐01, Beijing Emarbio Science & Technology Co., Ltd); and mouse anti‐β‐Actin monoclonal antibody (Cat. No. EM31011‐01, Beijing Emarbio Science & Technology Co., Ltd); then, the membranes were washed and probed with peroxidase‐conjugated goat anti‐mouse IgG (Cat. No. E030110‐01. EarthOx Life Science) or anti‐rabbit IgG (Beyotime). The bands were detected using an electrochemiluminescence system.

### 2.6 Electrophysiology

Acute thoracolumbar spinal cord slices (450 μm thickness) were prepared from 7‐ to 8‐week‐old rats using a vibratome (Leica VT1000S) as described previously (Yoshimura & Nishi, 1993; Zhao et al., 2017) and kept at 31°C for at least 30 min in artificial cerebral spinal fluid (ACSF) containing 95 mM NaCl, 1.8 mM KCl, 1.2 mM KH_2_PO4, 7 mM MgSO_4_, 0.5 mM CaCl_2_, 26 mM NaHCO_3_, and 15 mM D‐glucose, and 50 mM sucrose and aerated with 95% O_2_ and 5% CO_2_ (Zhao et al., 2017). For recording, each spinal cord slice was visualized under an infrared differential interference contrast optics microscope (Olympus). Whole‐cell patch clamp recordings were made at room temperature from SDH neurons in lamina II of the spinal cord. The neurons that produced delayed firing of action potential under current‐clamp conditions were selected for further experiments (Farrell et al., 2017; Ikeda, Heinke, Ruscheweyh, & Sandkühler, 2003); the data were collected with a MultiClamp 700B amplifier (Axon Instruments) and pCLAMP software (v.10.3, Axon Instruments) and digitized at 10 kHz (Digidata1322A, Axon Instruments). The spinal cord slice was continuously perfused with ACSF containing 127 mM NaCl, 1.8 mM KCl, 1.2 mM KH_2_PO4, 1.3 mM MgSO_4_, 2.4 mM CaCl_2_, 26 mM NaHCO_3_ and 15 mM D‐glucose and aerated with 95% O_2_ and 5% CO_2_. Pipette electrodes (4–8 MΩ) were filled with 133 mM K‐gluconate, 8 mM NaCl, 2 mM Mg·ATP, 0.3 mM Na_2_·GTP, 0.6 mM EGTA and 10 mM HEPES (pH 7.2–7.4). Only cells with a resting membrane potential of at least −60 mV and a stable series resistance or capacitance were included in the analyses. Neurons were clamped at −70 mV, and spontaneous excitatory postsynaptic currents (sEPSCs) were recorded for at least 5 min to establish stable baseline values. The perfusion medium was changed to 200 μM ANA‐12 for at least 10 min without altering the perfusion rate, then, sEPSCs were recorded for 5 min. Picrotoxin (100 μM) was present to reduce GABAergic contributions throughout the experiment. All drugs were purchased from Sigma Aldrich (USA). ANA‐12 and picrotoxin were dissolved in dimethyl sulfoxide to create stock solutions. Before each experiment, the stock solutions were diluted with ACSF to obtain the specific concentrations.

### 2.7 Statistical analysis

Data are presented as the mean ± *SEM*. The data for EMG responses to CRD were analysed with one‐way ANOVA. Western blotting data were analysed with two‐tailed independent *t*‐tests. For the animal visceral hypersensitivity data before and after ANA‐12 injection, paired *t*‐tests were used. The open field data were analysed using two‐tail independent *t*‐tests or Wilcoxon two‐sample tests. sEPSCs data were analysed using Clampfit 10.3 (Axon Instruments) and Mini‐analysis 6.0 (Synaptosoft Inc). The cumulative fraction of the amplitudes and the inter‐event intervals of the sEPSCs were compared using Kolmogorov–Smirnov tests; the 3 min period just before ANA‐12 application served as the control and was compared with the amplitudes or frequencies in a 3 min period beginning 10 min after the start of ANA‐12 application. Group means were compared using a paired *t*‐test. Statistical analysis was performed using GraphPad Prism 8.0. *p* < .05 was considered statistically significant.

## 3 RESULTS

### 3.1 The expression of BDNF and the TrkB receptor increased in NMS rats

**Figure 1:**
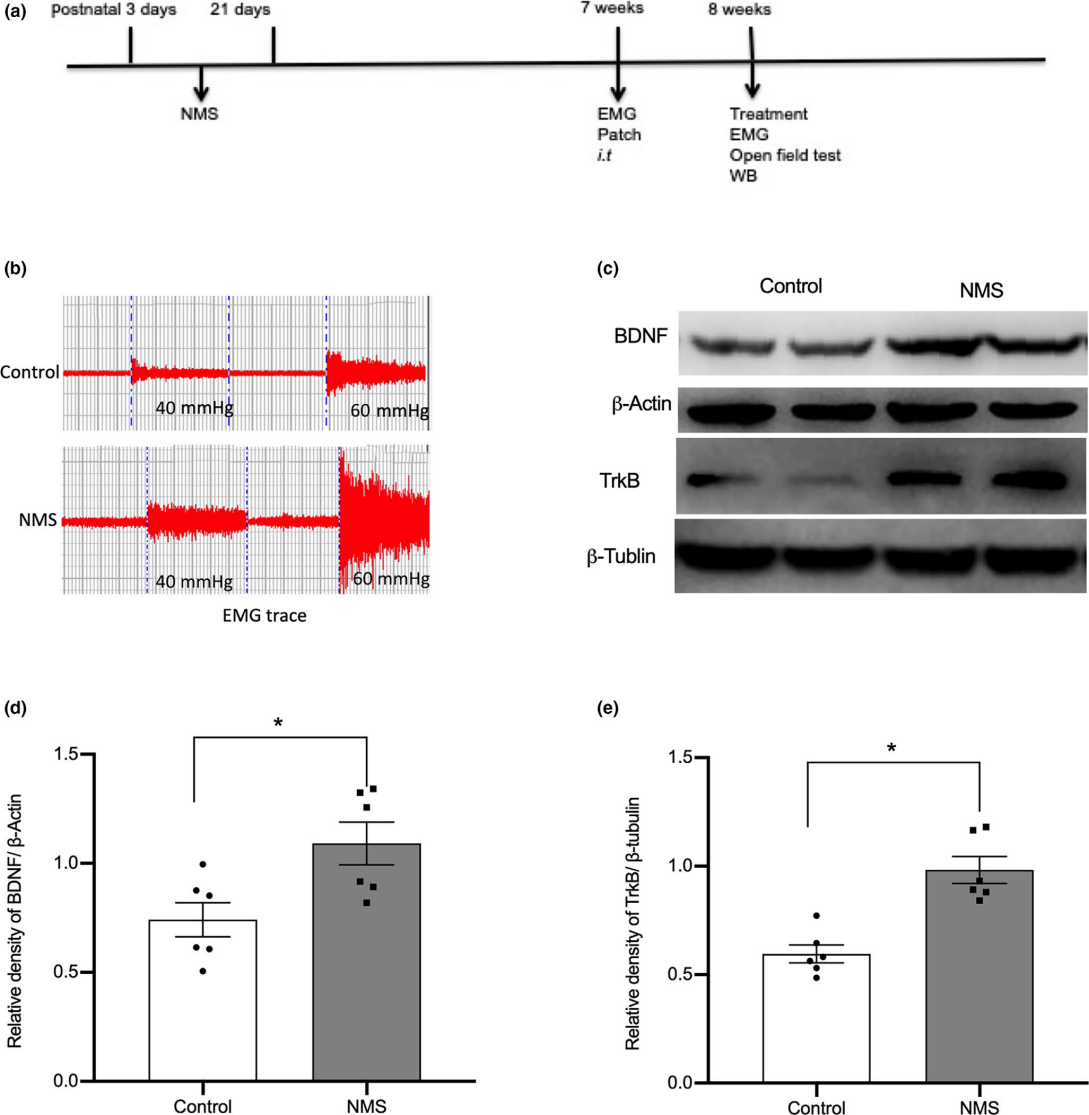
Assessment of visceral hypersensitivity and BDNF and TrkB expression in rats. **(**a) Timeline of the experimental design; (b) Representative traces of EMG recordings from control and NMS rats to assess visceral hypersensitivity; (c) Western blotting for the protein expression of BDNF and TrkB; (d and e) Bar graph of the protein expression of BDNF and TrkB normalized to β‐actin or β‐tubulin. *n* = 6, per group. *p < .05, compared to control rats

A timeline is shown to clarify the sequence of events in the experimental design (Figure 1a). After NMS, visceral hypersensitivity was assessed with the EMG response to CRD pressure and indicated that NMS caused visceral hypersensitivity in adult rats (Figure 1b). To determine whether NMS affected BDNF/TrkB, we examined the expression of BDNF and TrkB in thoracolumbar spinal segments. The protein expression of BDNF and TrkB increased in the NMS rats compared to the control rats (Figure 1c,d and e, *p* < .05, *n* = 6).

### 3.2 ANA‐12 attenuates visceral hypersensitivity in NMS rats

**Figure 2:**
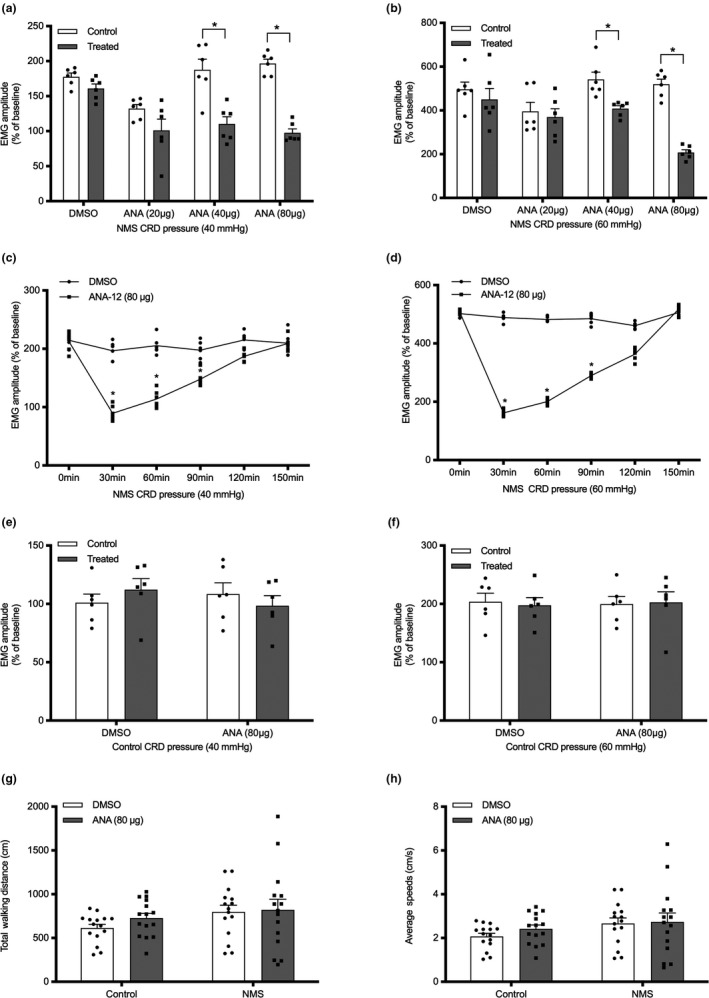
Effect of ANA‐12 in rats. (a and b) Bar graph of EMG amplitude 30 min after spinal intrathecal injection of DMSO or ANA‐12 (20, 40 or 80 μg) in NMS rats, with CRD of 40 and 60 mmHg, respectively; *n* = 6, per group; (c and d) Time curve of the effect of ANA‐12 (80 μg) at 40 and 60 mmHg CRD, respectively, in NMS rats; *n* = 6, per group; (e and f) Bar graph of EMG amplitude 30 min after spinal intrathecal injection of ANA‐12 (80 μg) in control rats; (g and h) Effects of ANA‐12 on total walking distance and the average speed, respectively, in control and NMS rats; *n* = 15, per group. *p < .05

To examine the effect of ANA‐12 on visceral hypersensitivity, we injected NMS rats intrathecally with different doses of ANA‐12. The EMG response to 40 and 60 mmHg CRD pressure was examined before and after intrathecal injection of ANA‐12 in NMS rats. ANA‐12 at 40 and 80 μg significantly suppressed the EMG response in NMS rats, however, the 20 μg dose had no effect (Figure 2a,b, *p* < .05, *n* = 6). Therefore, the 80 μg dose was used for the subsequent experiments. To further assess the time course of the effect of ANA‐12, we recorded the EMG response in NMS rats at intervals of 30 min after the administration of ANA‐12 (80 μg) for a total of 150 min. The lowest standardized amplitudes of the EMG response to 40 and 60 mmHg CRD were recorded 30 min after ANA‐12 injection (Figure 2c,d, respectively; *p* < .05, *n* = 6). The data indicated that the maximal inhibition was observed 30 min after the administration of ANA‐12 (80 μg) and gradually returned to normal over 150 min. Meanwhile, ANA‐12 (80 μg) had no significant influence on EMG responses in control rats (Figure 2e, *p* > .05, *n* = 6).

To determine the side effects of spinal intrathecal injections of ANA‐12 (80 μg) on spontaneous locomotor activity in rats, we conducted the open field test. ANA‐12 had no effect on walking distance or average speed (Figure 2f,g, respectively; *p* > .05, *n* = 15) in the NMS group compared to the DMSO group, suggesting that spinal intrathecal injections of ANA‐12 (80 μg) had no significant influence on spontaneous locomotor activity in rats.

### 3.3 The protein expression of PKMζ decreased after ANA‐12 administration in NMS rats

**Figure 3:**
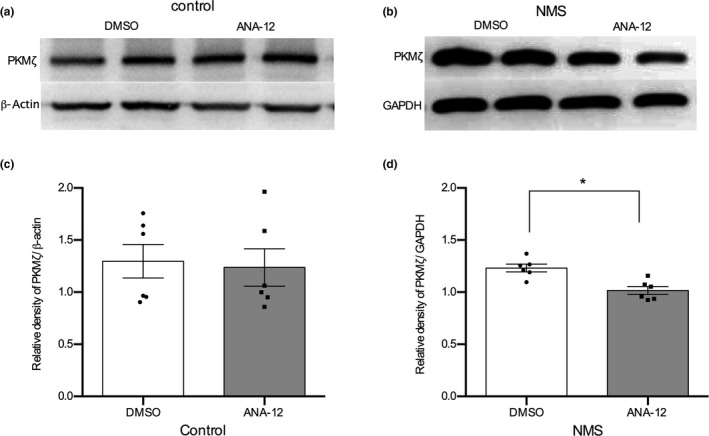
Effect of ANA‐12 on PKMζ protein expression in rats. (a) The protein expression of PKMζ after ANA‐12 administration in control rats. (b) The protein expression of PKMζ after ANA‐12 administration in NMS rats. (c) Bar graph of PKMζ protein normalized to the level of β‐actin after ANA‐12 administration in control rats. *n* = 6, per group. (d) Bar graph of PKMζ protein normalized to the level of GAPDH after ANA‐12 administration in NMS rats. *n* = 6, per group. *p < .05

Brain‐derived neurotrophic factor increases PKMζ protein expression at the spinal synapses in a chronic allodynia pain state (Melemedjian et al., 2013). We previously found that PKMζ or pPKMζ expression increased in the hippocampus (Chen et al., 2015) or the spinal segments of IBS‐like model rats (Tang et al., 2016). Furthermore, we found that BDNF expression was enhanced in NMS rats; is it possible that BDNF could regulate PKMζ expression? To determine whether PKMζ is regulated by BDNF/TrkB in NMS rats, we tested the protein expression of PKMζ after inhibiting the TrkB receptor with ANA‐12. After intrathecal injection of ANA‐12 (80 μg), the protein level of PKMζ decreased at the thoracolumbar spinal level in NMS rats (Figure 3b and d, *p* < .05, *n* = 6), but not in control rats (Figure 3a and c, *p* > .05, *n* = 6). These results suggested that ANA‐12 could suppress the expression of PKMζ in NMS rats.

### 3.4 The synaptic activity of SDH neurons was altered in NMS rats

**Figure 4:**
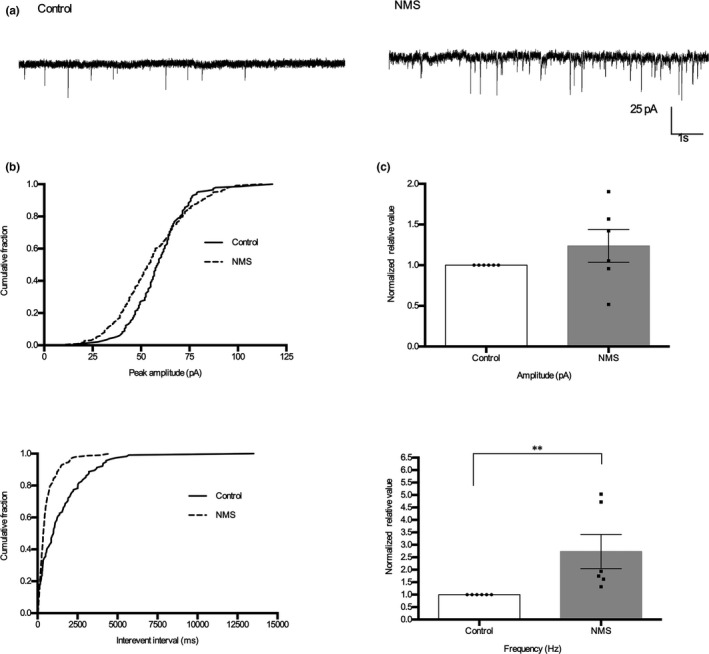
Synaptic activity of SDH neurons increased in NMS rats. (a) Representative traces of sEPSCs recordings from thoracolumbar spinal cord slices taken from control and NMS rats. (b) Cumulative fraction of peak amplitude and inter‐event interval of control and NMS rats. (c) Bar graph of amplitude and frequency normalized to the control rats. Neurons, *n* = 6, animals, *n* = 5, per group; **p < .01, *t*‐test

**Figure 5:**
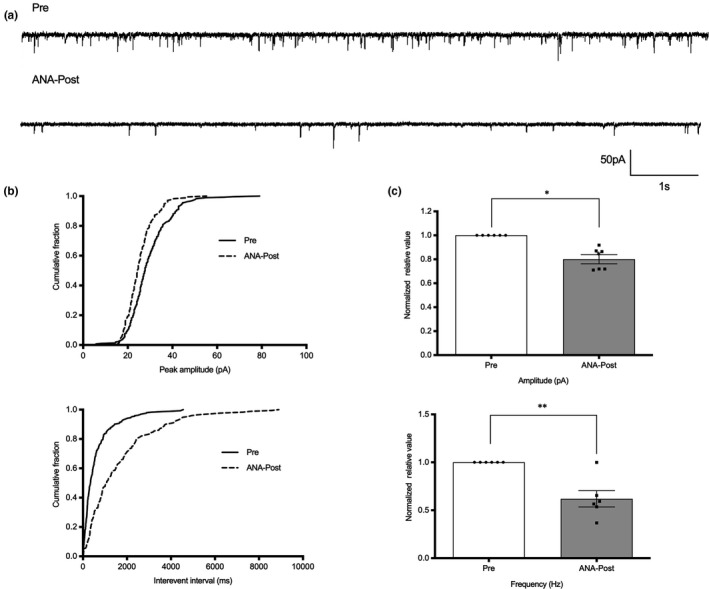
ANA‐12 reduced the synaptic activity of SDH neurons in NMS rats. (a) Representative traces of sEPSCs recordings from thoracolumbar spinal cord slices taken from NMS rats. (b) Cumulative fraction of peak amplitude and inter‐event interval before and after ANA‐12 applications. (c) Bar graph of amplitude and frequency normalized to the pre‐ANA‐12 baseline values in NMS rats. Pre: before ANA‐12 administration; ANA‐Post: after ANA‐12 administration; neurons, *n* = 6, animals, *n* = 5, per group; *p < .05, **p < .01, paired *t*‐test

To further examine synaptic activity in NMS rats and observe the effects of ANA‐12, we performed whole‐cell patch clamp recordings in thoracolumbar spinal slices from control and NMS rats. The frequency of sEPSCs was increased in NMS rats compared to control rats (Figure 4, *p* = .0015, *n* = 6). The amplitude and frequency of sEPSCs were reduced after perfusion with ANA‐12 (200 μM) for 10 min (Figure 5a,b). The amplitude of sEPSCs was reduced from −48.02 ± 6.09 pA without ANA‐12 to −37.57 ± 3.89 pA with ANA‐12 (20.41 ± 2.08% decrease, Figure 5c, *p* = .0134, *n* = 6). Additionally, the frequency of sEPSCs was reduced from 1.813 ± 0.53 Hz without ANA‐12 to 1.061 ± 0.29 Hz with ANA‐12 (40.75 ± 5.7% decrease, Figure 5c, *p* = .0067, *n* = 6). These results suggested that synaptic activity increased in NMS rats, and ANA‐12 could reduce the synaptic activity of SDH neurons in the spinal lamina II of the spinal cord in NMS rats.

## 4 DISCUSSION

In this study, we hypothesized that ANA‐12, an antagonist of TrkB, could be a candidate therapeutic agent for visceral hypersensitivity through the inhibition of BDNF/TrkB/PKMζ signalling in NMS rats. The expression level of BDNF/TrkB at the thoracolumbar spinal segments was enhanced in NMS rats. Additionally, ANA‐12 significantly suppressed visceral hypersensitivity without side effects on motricity. Finally, both synaptic activity and the expression of PKMζ in the thoracolumbar spinal segments significantly decreased after the administration of ANA‐12 in NMS rats.

### 4.1 BDNF/TrkB contributed to visceral hypersensitivity in NMS rats

Neonatal maternal separation triggered visceral hypersensitivity and dysfunction in several rat models (Chung et al., 2007; Hu et al., 2013). Our previous study (Chen et al., 2017) and this study further confirmed that NMS produced visceral hypersensitivity in rats.

Brain‐derived neurotrophic factor, a member of a small family of nerve growth factors, plays an important role in mediating long‐term changes in the synaptic proteome, synaptic plasticity and LTP (Horch & Katz, 2002; Leal, Comprido, & Duarte, 2014). An increased abundance of extracellular BDNF can enhance neuronal firing via proximal localization of the plasticity at the initial segment of the axon (Guo, Su, Chen, & Chai, 2017), even facilitating synapse elimination (Choo et al., 2017). BDNF contributes to spinal mechanical hyperalgesia (Li et al., 2017; Marcos et al., 2017) and the process of neuropathic pain (Smith, 2014).

Colonic BDNF expression is increased in IBS patients (Wang et al., 2015; Yu et al., 2012). Furthermore, research in BDNF^+/−^ mice has confirmed that colonic BDNF expression contributes to visceral hypersensitivity in IBS mice (Wang et al., 2016). BDNF mRNA and protein expression are also increased in the smooth muscle cells of a rat model of bowel obstruction (Lin, Fu, Radhakrishnan, Huang, & Shi, 2017). Apart from the increase in colonic BDNF expression, the expression of BDNF in the spinal cord and DRG is high in colitis (Qiao, Gulick, Bowers, Kuemmerle, & Grider, 2008). These prior findings, together with our study, show that overexpression of BDNF not only in the colon level but also on the spine can contribute to visceral hypersensitivity.

Nevertheless, another question must be addressed: how does BDNF contribute to high visceral hypersensitivity in NMS rats? We hypothesized that BDNF would recruit its high‐affinity receptor TrkB (Klein et al., 1991; Soppet et al., 1991) in the thoracolumbar spinal segments in NMS rats; therefore, we performed western blotting to measure the expression of the TrkB receptor and found it to be significantly increased in NMS rats. This result shows that the BDNF/TrkB signalling pathway might be implicated in the formation of visceral hypersensitivity in NMS rats. However, BDNF is expressed in different cellular populations throughout the nervous system, such as neurons, astrocytes and microglia, and is involved in different forms of plasticity (Ferrini & De Koninck, 2013; Hedrick et al., 2016). Among the cell types that release BDNF in the spinal cord, which ones play a dominant role in the formation of visceral hypersensitivity? Further research should be performed in the future.

### 4.2 ANA‐12 attenuates visceral hypersensitivity by inhibiting BDNF/TrkB signalling in the spinal cord of NMS rats

ANA‐12 has been characterized as a selective TrkB antagonist (Cazorla et al., 2011) and its use has been reported in studies of BDNF/TrkB signalling (Azogu & Plamondon, 2017; Barnes, Koul‐Tiwari, Garner, Geist, & Datta, 2017). In our studies, ANA‐12 was administered by intrathecal injection in NMS rats to determine the appropriate dosage. From the time‐response curve of different doses of ANA‐12, we observed that a dose of 80 μg 30 min effectively relieved visceral hypersensitivity in NMS rats but not in control rats. Furthermore, the open field results showed that spinal intrathecal injections of ANA‐12 (80 μg) had no significant influence on spontaneous locomotor activity in rats, which is similar to the findings of Cazorla (Cazorla et al., 2011). Burgos‐Vega also found that intraperitoneal injections of ANA‐12 (0.5 mg/kg) blocked allodynia in IL‐6‐treated rats (Burgos‐Vega et al., 2017). Furthermore, a recent study reported that blocking the BDNF/TrkB signalling pathway reversed injury‐induced pain hypersensitivity at the spinal dorsal horn (Echeverry et al., 2017). A BDNF/TrkB‐directed drug was also discovered to induce synaptic dysfunction in a growth factor‐driven expansion and inhibition of notch (GRINCH) neuron model (Traub et al., 2017). When we applied ANA‐12 to block BDNF/TrkB signalling, visceral hypersensitivity was suppressed in NMS rats. Our study also confirmed that inhibition of the BDNF/TrkB signalling pathway with ANA‐12 could attenuate visceral hypersensitivity in NMS rats. Thus, previous findings in combination with the present results suggest the BDNF/TrkB signalling pathway as a prospective therapy target in male rats. However, recent research (Mapplebeck et al., 2018) reported the existence of sexually dimorphic pain signalling in rats. In the future, it could be interesting to explore the sex difference in BDNF/TrkB signalling in visceral hypersensitivity.

### 4.3 ANA‐12 decreased the expression of spinal PKMζ and reduced synaptic activity in NMS rats

Structural plasticity and the reorganization of synapse cells and circuits contribute to chronic pain (Kuner & Flor, 2016). PKMζ is a crucial signalling kinase that regulates central hypersensitivity and sustains spinal nociceptive plasticity in inflammation and neuropathic pain (Asiedu et al., 2011; Laferrière et al., 2011; Li et al., 2010). PKMζ expression increased in the ACC of rats with inflammation pain, while inhibition of PKMζ relieved pain (Du et al., 2017). We previously found that PKMζ or pPKMζ expression increased in the hippocampus (Chen et al., 2015) or the thoracolumbar spinal segments of IBS‐like model rats (Tang et al., 2016), which indicated that spinal PKMζ contributes to visceral hypersensitivity.

BDNF is an important mediator of pain in the dorsal horn, as mentioned previously. The maintenance of late LTP by BDNF was abolished via the inhibition of PKMζ (Mei, Nagappan, Ke, Sacktor, & Lu, 2011). BDNF increases PKMζ protein expression at the spinal synapses in chronic allodynic pain states (Melemedjian et al., 2013). These studies showed a potential link between BDNF and PKMζ. In our experiments, the expression of PKMζ decreased after the intrathecal application of ANA‐12 in the NMS rats, indicating that BDNF/TrkB partly regulated visceral hypersensitivity via PKMζ. Nevertheless, another question must be addressed: how does BDNF/TrkB regulate PKMζ to contribute to visceral hypersensitivity in NMS rats? One hypothesis is that BDNF recruits PKMζ proteins to synaptic sites to potentiate synaptic responses. BDNF may regulate the translocation of PKMζ from the cytoplasm to the synaptic sites (Mei et al., 2011).We previously found that inhibiting GluA2‐containing AMPARs could alleviate visceral hypersensitivity in IBS‐like model rats (Chen et al., 2017). Helfer presented a computational model showing the interlinked feedback loops of PKMζ and GluA2‐containing AMPARs (Helfer & Shultz, 2018). In our present study, the whole‐cell patch clamp recordings also showed that the frequency of sEPSCs was increased in NMS rats. Furthermore, we found that the amplitude and frequency of sEPSCs were reduced after inhibition of TrkB in NMS rats, which demonstrated that synaptic activity was decreased. The evidence suggests the possibility that BDNF/TrkB regulates PKMζ and GluA2‐containing AMPARs, potentiating synaptic responses to contribute to visceral hypersensitivity in NMS rats. However, the interactions between TrkB and PKMζ still need to be further investigated. Tandem protein immunopurification should be performed in the future.

In conclusion, NMS caused visceral hypersensitivity and increased sEPSCs in adult rats by activating the BDNF/TrkB/PKMζ in the thoracolumbar spinal cord. ANA‐12, by blocking the action of the BDNF/TrkB pathway, could potentially act as a therapeutic drug for visceral hypersensitivity in IBS patients.

## CONFLICTS OF INTEREST

None declared.

## AUTHOR CONTRIBUTIONS

F.F. and C.L. designed research. F.F., Y.T. and H.D. performed research. F.F., Y.T., Y.C. and P.S. contributed to acquisition of data. F.F., Y.C. and A.C. analysed data. F.F. and C.L. wrote the paper.
